# Loneliness From the Digital Mental Health Practitioners’ Perspective: Thematic Analysis of Semistructured Interviews

**DOI:** 10.2196/89798

**Published:** 2026-07-07

**Authors:** Gregor Milligan, Liz Dowthwaite, James Goulding, Elvira Perez Vallejos

**Affiliations:** 1 School of Computing and Digital Technologies, College of Business, Technology and Engineering, Sheffield Hallam University, Sheffield, England, S1 1WB, United Kingdom, +44(0)7795660354; 2 N/LAB, Nottingham University Business School, University of Nottingham, Nottingham, England, United Kingdom; 3 School of Computer Science, University of Nottingham, Nottingham, England, United Kingdom; 4 School of Medicine, University of Nottingham, Nottingham, England, United Kingdom

**Keywords:** loneliness, stigma, digital mental health, practitioner perspective, thematic analysis

## Abstract

**Background:**

Loneliness is a prevalent concern across the United Kingdom. While validated scales exist to quantify the severity of loneliness across populations, there remains a gap in understanding how loneliness manifests and is addressed within therapeutic practice. Given the associated stigma surrounding loneliness, practitioner perspectives offer crucial insights into how clients express loneliness within digital therapeutic environments. These insights can inform more nuanced conceptualizations of loneliness.

**Objective:**

This study aimed to gather the practitioners’ perspectives on loneliness within a digital therapeutic context and were defined as follows: (1) understand how practitioners identify loneliness concerns, (2) identify how loneliness is elicited in digital mental health interventions, and (3) identify co-occurring themes (such as grief, shame, and social disconnection) that signal loneliness concerns in client communications within digital therapeutic environments.

**Methods:**

Semistructured interviews were conducted with 9 practitioners. Participants included specialists in grief counseling, lesbian, gay, bisexual, transgender, and queer or questioning plus support; and digital mental health therapists. Interview transcripts were analyzed using thematic analysis, using an inductive, data-driven approach to allow themes to emerge from participant accounts rather than fitting data to preexisting theoretical frameworks.

**Results:**

The following four themes were identified: (1) Conceptualizing Loneliness: practitioners distinguished between social contact and meaningful connection; (2) Contextual Causes: loneliness emerged from life transitions, stigmatized identities, and resource reduction (eg, youth services closures and social support); (3) Expressions and Language: clients rarely expressed loneliness directly, instead using proxy terms, with disclosure patterns varying by age; and (4) Mental Health Co-occurrence: severe mental health conditions created bidirectional cycles of loneliness, exacerbated by symptoms of mental health difficulties. Practitioners reported that many clients experienced loneliness concerns, yet direct disclosure was absent across all participants’ experiences.

**Conclusions:**

Practitioners identified multiple stigmatizing experiences as contextual drivers of loneliness, particularly demonstrating how loneliness emerges not only from individual experiences but from broader patterns of social exclusion and marginalization. For therapeutic practice, these insights suggest that practitioners can use awareness of stigmatizing experiences as potential indicators when assessing loneliness risk. The presence of contextual patterns was consistent across practitioners’ experiences, providing a foundation for developing more targeted interventions to address both the emotional experience of loneliness and the underlying social drivers.

## Introduction

### Overview

Loneliness is a growing concern in the United Kingdom, with its prevalence increasing year over year [1]. The Campaign to End Loneliness published data showing that 49.63% of UK adults report feeling lonely often or sometimes [2]. This prevalence is seen internationally, with 40% of older adults in the United States reporting feeling lonely [3] and 37.9% for Chinese adults aged older than 50 years [4]. Understanding this concern at an individual level is pertinent as loneliness demonstrates bidirectional relationships with other mental health concerns [5], whereby loneliness both increases vulnerability to mental health difficulties and emerges as a consequence of them.

While numerous validated scales exist to quantify loneliness experiences across populations (eg, the University of California, Los Angeles [UCLA] Loneliness Scale and the De Jong Gierveld [DJG] Loneliness Scale) [6,7], there remains limited research examining how loneliness manifests within therapeutic practice from practitioners’ perspectives, particularly in digital mental health contexts.

Given the relationship between loneliness and co-occurring mental health conditions [8,9] and the interpersonal stigma that may affect therapeutic disclosure [10,11], understanding how experienced practitioners identify and interpret loneliness presentations is crucial for informing therapeutic practice and intervention approaches.

With the advent of digital mental health interventions, those in need have more ready access to cost-effective and context-appropriate therapy. Online interventions can be broad or targeted, with numerous services for anxiety, depression, and suicide prevention [12-14]. There are also broader interventions such as internet-based cognitive behavioral therapy [15] and text-based talking therapies [16].

As digital mental health platforms become increasingly prominent points of therapeutic contact [12], questions arise about how loneliness manifests and is communicated within these environments. Practitioners working in these contexts may observe distinct patterns in how clients express concerns through text-based interactions, or alternatively, expression may remain consistent across therapeutic modalities. Understanding practitioners’ perspectives from these digital environments provides an opportunity to examine loneliness disclosure patterns in contemporary therapeutic practice and explore how interpersonal stigma operates within online therapeutic relationships.

This study focuses on practitioner insights through a thematic analysis (TA) of experienced practitioners in digital therapeutic practice. This study’s aims were the following: (1) understand how practitioners identify loneliness concerns, (2) identify how loneliness is elicited in digital mental health interventions, and (3) identify co-occurring themes (such as grief, shame, and social disconnection) that signal loneliness within digital therapeutic environments.

These aims enable the exploration of the following question: How do digital mental health practitioners provide insight into loneliness in digital therapeutic contexts? Furthermore, this study seeks to develop a comprehensive understanding of how loneliness is directly and indirectly expressed by clients in digital mental health settings through practitioner interviews.

### Background

The growth of digital mental health interventions has created a substantial cohort of practitioners with experience in identifying and responding to loneliness concerns across diverse client populations. Practitioners occupy a unique observational position in loneliness research. While individual clients may experience loneliness, practitioners encounter multiple cases over time, enabling the identification of patterns in disclosure, the recognition of co-occurring themes, and the observation of how loneliness manifests in therapeutic contexts. This accumulated clinical expertise represents a valuable but underexplored data source for understanding how loneliness is expressed, experienced, and addressed in contemporary digital therapeutic practice.

While there are quantitative elucidations into the co-occurring factors of loneliness [17,18], qualitative evidence can offer nuanced experiences that a loneliness survey or measure cannot achieve due to an individual’s inability to describe their lived experience.

### Qualitative Insights Into Loneliness

There have been several qualitative studies examining the experience of loneliness; however, they are primarily from the lonely individual’s perspective. Such studies are often stratified by a specific demographic characteristic [19]. Although there are nuances across these demographics, similar trends have emerged, such as the importance of connectedness to the experience of loneliness [20]. Studies have shown a U-shaped distribution across age, indicating that younger and older individuals experience loneliness more frequently than those in middle age [19,21,22].

Qualitative research has identified consistent experiences that can be used to identify the core characteristics of loneliness. Martin et al [23] interviewed 33 adolescents stratified by socioeconomic area, finding that young people identified two core elements defining their loneliness experience: (1) connectedness with friends and (2) perceptions of “aloneness.” Fardghassemi and Joffe [24] used a free association technique and TA with 48 young adults (aged 18‐24 years) from the four most deprived boroughs of London, identifying the following five key themes explaining the subjective causes of loneliness: (1) The Feeling of Being Disconnected, arising from feeling one does not matter, is not understood, or is unable to express oneself; (2) Contemporary Culture, relating to social media and materialism; (3) Pressure, associated with finding work or friends; (4) Social Comparison; and (5) Transitions Between Life Stages, including the breakdown of relationships and transitions in education or employment. These identified themes offer a subjective overview of loneliness, which warrants further specific contextual analysis.

Loneliness is highly contextual and often related to a number of co-occurring facets of a person’s life. For example, Verity et al [25] evaluated how young people conceptualize loneliness across age ranges through qualitative interviews with 24 young people aged 8‐14 years. They found that young people conceptualize loneliness differently in different contexts, such as the experience of loneliness at school compared with loneliness at home. Verity et al [25] also reinforced the idea that loneliness cannot be attributed to a single cause but varies across the sample, such as change or social exclusion from peer groups, causing increased loneliness. These identified experiences offer an overview of the subjective experience of loneliness but do not address how these experiences are communicated or identified within therapeutic practice.

Themes of connectedness and social comparison are present across different age groups and study contexts. Both studies by Fardghassemi and Joffe [24] and Martin et al [23] highlight that loneliness fundamentally stems from a perceived lack of meaningful connection rather than just being alone, suggesting that regardless of specific demographic characteristics or qualitative approaches, the loneliness experience centers on unmet needs for meaningful social connections. While these contextual concerns do offer some insight into loneliness, stigma serves as a significant overarching experience of an individual’s lived experience, which directly exacerbates feelings of loneliness.

### Loneliness and the Impact of Stigmatized Experiences

Stigma is of particular note as it leads to challenges in both social and therapeutic environments, where an individual may not be as open to discussing a concern due to the stigmatized nature of either the antagonist of loneliness or loneliness itself. Barreto et al [10] analyzed loneliness and stigma across several determinants, finding that the stigma *of* loneliness significantly affects disclosure patterns, with one third of UK respondents reporting that they would be embarrassed to admit feeling lonely, and quantitative loneliness measures producing higher scores when avoiding direct loneliness terminology. Barreto et al [10] demonstrated that stigma drives concealment behaviors, with women more likely to report shame when experiencing loneliness, while men perceived greater community stigma around loneliness, providing support for why clients might avoid direct loneliness expression in therapeutic settings. There is a link between experiences of shame and loneliness [26,27]. The way in which mental health concerns are discussed in the media also contributes to their general stigmatization [28] as they are seen to be negative experiences. People with mental illness are presented in the media as “peculiar,” “different,” and “dangerous” [29], providing a narrative which perpetuates sensationalized and stigmatizing characterizations [30].

Crucially, it has been established that those who experience loneliness feel shame because they are lonely; Dahlberg [31] evaluated interviews with participants aged between 11 and 82 years, who described the experience of loneliness as “shameful” and “ugly.” Loneliness can perpetuate from feelings of shame (rather than only causing shameful feelings) for those who have been othered in some way [11], such as those who experience rejection and neglect from peers, who may feel particularly stigmatized [10,27,32]. Madsen et al [33] conducted 15 semistructured individual interviews with adolescents aged 14‐15 years who were immigrants, descendants, or had a Danish majority background, finding that loneliness was often described as a challenging feeling that varied in intensity and duration. Crucially, Madsen et al [33] characterized loneliness as an “invisible social stigma,” in which loneliness itself is a stigmatized experience. Stigma as a driving force of loneliness may therefore shape how individuals disclose loneliness within therapeutic contexts, representing a gap in the broader literature that practitioner perspectives are uniquely positioned to address.

Mental health is a stigmatized experience that often co-occurs with loneliness [34-36], and understanding the relationship between this specific preexisting stigma and loneliness is particularly critical.

### Loneliness Co-Occurrence With Mental Health Concerns

The idea of being “othered” is a factor in the experience of mental health concerns; in turn, those who experience significant or severe mental health episodes, such as psychosis, are often lonely due to this isolating experience and the stigma around these concerns [9,36,37]. There is an established relationship between loneliness and mental health concerns. Birken et al [38] interviewed people who had self-reported mental health concerns to establish connections between loneliness and mental health, identifying contributory factors to continuing loneliness and approaches for reducing loneliness.

While there are overlaps between loneliness and depression, understanding how practitioners differentiate these experiences in clinical practice requires observing the ways clients express loneliness within therapeutic environments. While individual lived experience remains crucial for understanding loneliness, practitioners’ perspectives offer a unique viewpoint, as their exposure to multiple loneliness presentations enables them to identify patterns that may not be apparent from individual accounts alone.

### Practitioners' Perspectives on Loneliness

Without practice-based insight from practitioners, the way in which loneliness is expressed and the experiences therein remain opaque. However, few qualitative interview studies of practitioners have been undertaken.

Stefanidou et al [39] interviewed 20 mental health practitioners who focus on first-episode psychosis in the United Kingdom; they reported that the majority of service users with early psychosis experience feelings of loneliness. They often encountered socially isolated and disconnected clients, believing them to be lonely, but service users rarely discussed loneliness explicitly in clinical interactions.

Dobarrio-Sanz et al [40] interviewed 26 health care professionals about their perceptions of loneliness in older adults, identifying that loneliness is highly contextual and should be taken more seriously as a public health concern. Verity et al [41] analyzed online counseling conversations between Childline counselors and adolescents, finding that issues with trust and self-worth acted as significant barriers to help-seeking, consistent with the broader pattern of stigma-driven concealment of loneliness concerns.

These insights reinforce the consistent pattern across age groups and clinical contexts that loneliness fundamentally centers on peer relationship quality and social environmental pressures, regardless of age group or other demographic characteristics.

While not specifically mental health practitioners, Jovicic and McPherson [42] interviewed general practitioners (GPs), finding that those experiencing loneliness visit their GP more frequently than those who are not, which has the potential to put a strain on GP and health support waiting lists while also increasing costs. They also determined that loneliness co-occurs with physical and mental health conditions while affecting cognition and lifestyle.

There are consistent themes around connectedness and stigma that emerge from studies with both individuals and practitioners. Practitioner insights are rarely examined; however, when they are, they reveal ways in which individuals experience loneliness [39,40,43]. For example, there is specific language used by those who experience loneliness and disclose it to practitioners. Understanding the language used to disclose loneliness can provide practitioners with context for identifying when a client is expressing loneliness and offer some understanding of the nuances of the experience.

This study addresses the gap in limited practitioner-based conceptualization, in which practitioners describe their experiences of engaging with clients in several contexts and provide much-needed elucidation and evidence regarding loneliness in therapeutic practice. Practitioners can also observe longitudinal patterns and changes across treatment periods, providing contextualization of challenges that individual client interviews might overlook. This opportunity to recognize patterns across cases represents an opportunity to enhance our understanding of loneliness, which is unavailable through studies of single instances of loneliness.

While this preceding body of work (summarized in Table 1) demonstrates the co-occurring themes based on practitioner expertise, there remains very limited qualitative research focusing specifically on practitioners within digital mental health interventions. This is a notable gap given the growing uptake of online mental health support, and the unique ways in which loneliness might be expressed and addressed in digital contexts. This study addresses this gap by examining how digital mental health practitioners perceive and respond to loneliness in their practice.

**Table 1.** Summary of practitioner interviews on loneliness in health care and mental health.

Study	Setting (country)	Participants	Method	Key findings
Stefanidou et al [39]	Early intervention services for first-episode psychosis (United Kingdom)	20 mental health practitioners (multidisciplinary team)	Semistructured interviews	Loneliness is common in service users, linked to isolation and disconnection, and rarely discussed explicitly in sessions.
Dobarrio-Sanz et al [40]	Health care for older adults (Spain)	26 health care professionals (nurses, physicians, and psychologists)	Interviews and focus groups	Loneliness perceived as highly contextual, underrecognized, and requires a public health focus.
Jovicic and McPherson [42]	Primary care (United Kingdom)	19 general practitioners	Semistructured interviews	Loneliness is linked to higher general practitioner visits, co-occurs with mental or physical conditions, and impacts cognition and lifestyle.
Lawn et al [43]	Mental health services (Australia)	322 survey respondents (consumers, carers, and practitioners)	Survey (mixed sample and limited practitioner-specific reporting)	Loneliness is linked to stigma, judgment, and lack of safe spaces; practitioner perspectives are not clearly separated.
Verity et al [41]	Childline online counseling (United Kingdom)	Childline counselors (analysis of transcripts)	Qualitative TA^a^ of chat transcripts	Five themes, 12 subthemes; young people use short-term coping strategies; and trust and self-worth are key barriers.
This study	Digital mental health platforms (United Kingdom)	9 digital mental health practitioners (grief counseling, LGBTQ+^b^ specialists, and platform moderators)	Semistructured interviews with the TA	High prevalence of loneliness perceived by practitioners, indirect expression only, stigma as a barrier, contextual causes, and co-occurrence with mental health conditions.

a TA: thematic analysis.

b LGBTQ+: lesbian, gay, bisexual, transgender, and queer or questioning plus.

## Methods

### Overview

TA of practitioner interviews was selected as the methodological approach, as it enables a granular view of loneliness across digital practice. Therefore, practitioners who practice digitally were recruited to answer a series of questions about their clients’ experience of loneliness. The scope of this paper is focused on practitioners’ experiences and perceptions of loneliness among their service users.

### Recruitment and Sample Characteristics

Nine practitioners participated in this study (see Table 2). Of these, 6 identified as female and 2 as male; 1 did not report gender. Participants ranged from 37 to 71 years, with 2 participants not reporting their age. All participants who reported ethnicity identified as White British, with 2 participants not reporting ethnicity. Participants were distributed across three digital mental health platforms: Kooth (n=3), Togetherall (n=2), and the Association for Counselling and Therapy Online (ACTO; n=4). The specific professional roles spanned grief therapy, emotional well-being practice, psychotherapy, mental health nursing, and dialectical behavior therapy, reflecting a range of clinical specialties and therapeutic approaches within digital mental health practice.

**Table 2.** Participant demographics and professional characteristics (n=9). All participants identified as White British except participants 3 and 6, whose ethnicity was not reported.

Participant	Sex	Age (years)	Professional role	Platform
P1	Female	71	Grief therapist (psychodynamic)	Association for Online Therapists and Remote-based therapy
P2	Female	40	Emotional well-being practitioner	Kooth
P3	Not reported	Not reported	Emotional well-being practitioner	Kooth
P4	Male	38	Dialectical behavior therapist and supervisor	Association for Online Therapists & Remote-based therapy
P5	Female	52	Counsellor and mental health mentor	Association for Online Therapists & Remote-based therapy
P6	Female	—	Emotional well-being practitioner	Kooth
P7	Female	59	Psychotherapist	Association for Online Therapists & Remote-based therapy
P8	Male	41	Wellbeing moderator	Togetherall
P9	Female	37	Mental health nurse and well-being practitioner	Togetherall

### Overview of Digital Intervention Platforms and Counseling Associations Recruited From

#### Kooth

Kooth is a UK-based digital mental health service commissioned by the UK National Health Service, local authorities, and third-sector organizations. Kooth provides free, confidential, and anonymous support to children, young people, and adults. The platform combines asynchronous features (eg, mood tracking and peer forums) with synchronous text-based counseling delivered by practitioners.

#### Togetherall

Togetherall is a peer-to-peer digital mental health platform accessible 24/7 and designed to offer anonymous support through community interaction. Membership is typically provided via institutions such as employers or health care providers.

### The ACTO

The ACTO is a UK professional membership organization that sets ethical and competency standards for practitioners delivering therapy remotely. ACTO maintains a directory of accredited online therapists and provides specialist training guidance functioning as a regulatory reference point and a professional support network for practitioners working in digital contexts.

### Materials and Procedures

Semistructured interviews were conducted using questions based on established literature to gain specific insights into the lived experience of loneliness and the language often used when disclosing concerns about loneliness. The interview schedule included the following questions:

1. How would you define loneliness?
2. How prevalent is the issue or concern of loneliness in your experience working with (their platform or digital therapeutic environment)?
3. What is your experience working with young people who experience loneliness?
4. What words and phrases are typically used when talking about loneliness in this context?
5. What is your experience working with young people who experience loneliness?

### Analysis Approach

TA, an iterative method was used to evaluate qualitative data [44]. Through TA, interview content was classified into broad themes, primarily driven by predefined interview questions. The semistructured nature of the interviews allowed the interviewer and practitioners to discuss tangential yet conceptually related themes that emerged during the interview. Themes were refined as the interviews were conducted, enabling the researcher to identify the point at which saturation of relevant opinions appeared to occur. Analysis used an inductive approach, allowing themes to emerge from participants’ accounts rather than fitting data into preexisting theoretical frameworks.

Analytic decisions during theme development were guided by the frequency and consistency of codes across the interview sample. Core themes were established as they recurred across the interviews, with codes appearing in only one or two interviews either absorbed into broader themes where conceptual overlap existed or excluded where codes represented contextually specific rather than generalizable themes. Coding and initial theme development were conducted by the lead researcher, who was also the interviewer, with themes subsequently reviewed and discussed with the wider research team to support analytical rigor.

Data saturation was approached pragmatically, recognizing that complete theoretical saturation is rarely achievable in qualitative research [45]. After nine interviews, the core thematic structure had been established, with no new primary themes emerging.

TA was performed using the following 6-phase approach outlined by Braun and Clarke [46]:

1. Familiarization and refamiliarization with the data: there was already a baseline of familiarization with the transcripts, as the researcher was also the interviewer. The generated transcriptions were captured via Microsoft Teams, then relistened to, allowing the transcripts to be corrected and refamiliarizing the researcher with the interview content.
2. Generation of initial codes: the transcripts were classified using codes, and new codes were generated if appropriate to encapsulate a new concept. During the first pass, the coding process was systematic yet flexible, allowing for the introduction of new concepts from each transcript. This was accomplished through the use of Lumivero’s NVivo (version 15), a tool used to evaluate transcripts and generate codes across a corpus.
3. Establishment of core themes: after being generated, codes were collated into initial themes to be further analyzed and evaluated.
4. Review of themes: an analysis of established themes was undertaken to identify gaps and overlaps. Overarching themes and subthemes were identified. Some themes were merged where overlap was present, while others were split to better capture distinct concepts.
5. Naming and definition of themes: the final themes were defined based on their overarching content and given appropriate names.
6. Report production: to support the reporting of the results, compelling examples were extracted to illustrate the themes identified in this analysis. The themes are discussed and contextualized with the other identified themes to generate conclusions about the findings.

### Researcher Positionality

The lead researcher approached this study from a multidisciplinary background in computing and psychotherapy research. To develop contextual familiarity with therapeutic practice, engagement with counseling literature and practice was undertaken before data collection. Analysis was conducted by a single coder, and the themes were evaluated by the wider research team. It is acknowledged that the interpretive process was informed by a prior theoretical assumption consistent with Hawkley and Cacioppo’s framework [20], namely, that loneliness reflects a discrepancy between desired and actual social connection. This assumption is made transparent here as it may have shaped the salience of certain themes during coding.

### Ethical Considerations

Ethical approval for this study was granted by the University of Nottingham Business School (application ID 202324037). The practitioner participants provided written informed consent when participating in the conducted interviews. Participants’ data has been anonymized in line with the University of Nottingham research ethics guidelines.

## Results

### Overview

TA of the semistructured interview transcripts revealed several interconnected themes related to the experience of loneliness. Based on transcript coding, the following four core themes were identified (see Table 3): (1) Conceptualizing Loneliness, (2) The Contextual Factors and Causes of Loneliness, (3) the Expressions and Language of Loneliness, and (4) The Co-occurrence of Loneliness and Mental Health Concerns. These themes directly address the research aims to understand how practitioners identify loneliness, its prevalence and manifestations in digital mental health interventions, and the expressions and co-occurring concerns that signal loneliness.

**Table 3.** Overview of core themes and subthemes.

Core themes and subthemes	Description
Conceptualizing Loneliness	
Loneliness Is a Lack of Meaningful Connection With Others	How practitioners define loneliness in terms of unmet connection and the quality of interpersonal relationships.
Loneliness vs Solitude	Practitioners' distinction between chosen solitude and unwanted isolation.
Loneliness Prevalence	Practitioners’ observations regarding the pervasive nature of loneliness.
Contextual Factors and Causes of Loneliness	
Transition, Loss, and Loneliness	How life transitions and bereavement contribute to loneliness experiences.
Stigma and Social Barriers	The role of stigma, discrimination, and social exclusion in creating and perpetuating loneliness.
The Reduction in Social Resources and Increased Cost of Living	How economic pressures and reduced social infrastructure contribute to loneliness.
The Expressions and Language of Loneliness	
Indirect Expressions of Loneliness	How service users communicate loneliness concerns without explicit disclosure.
Age-related Differences in Loneliness Expression	Variations in how different age groups articulate and present loneliness concerns.
The Co-Occurrence of Loneliness and Mental Health Concerns	
Pathways to Severe Mental Health Issues	How loneliness contributes to the development or exacerbation of mental health conditions.
Loneliness as a Consequence of Specific Mental Health Concerns	How certain mental health conditions create or intensify experiences of loneliness.

### Theme 1: Conceptualizing Loneliness

#### Overview

This theme captures how loneliness is conceptualized by practitioners, identifying a lack of connection with others as a fundamental component of loneliness. Practitioner 3 defined loneliness as

a feeling of wanting connection but having that not be fulfilled for some reason or another.

This idea of connection to others helps to differentiate loneliness from social isolation, in which someone can be connected with others but still feel lonely. It was further established that connections must be meaningful and supportive, Practitioner 9 described loneliness as

The absence of support and meaningful connection [...] No one understands who you truly are like the core self.

This highlights how loneliness is not only based on social experience but rather on an unmet social need.

The need for meaningful connection is demonstrated by practitioner 4, who identified the typical expression of loneliness:

I don’t feel like anyone understands me or I don’t feel like they are my actual friends.

This indicates how individuals can have a social group with which they engage, but without what they perceive to be a meaningful connection. Practitioner 3 highlights a core challenge for those experiencing loneliness:

No one’s doing anything wrong, but they still just don't quite connect.

This presents a challenge for both clients and practitioners in identifying clear contributing factors because it suggests loneliness can occur even in ostensibly functional relationships, making it difficult to attribute loneliness to specific relationship problems or social circumstances.

This feeling of belonging and connection with others is fundamental to the nature of loneliness as it drives feelings of shame. Practitioner 7 identifies the impact of shame on disclosure:

Often loneliness is a word I think people don’t always want to use. There’s an element of shame. Especially if they’ve got lots of friends, like, why do I feel lonely?

When relationships appear functional yet a meaningful connection remains absent, clients may question why they feel isolated despite having social relationships.

#### Loneliness Versus Solitude

A distinction emerged between loneliness and solitude; while loneliness was characterized as a negative experience, practitioner 2 described expressions of comfort in solitude:

There’s a lot of people who actually do well and really thrive in having that space and time alone and don’t seek that connection.

Solitude is the conceptual antithesis of loneliness, further illustrating the nuance of loneliness self-disclosure in which being alone does not necessarily equate to feeling lonely.

The idea of being “lonely in the crowd,” in which someone has a social network but feels that they lack a meaningful connection, is also present. Practitioner 4 identifies

this idea that you can have many people surrounding you but that feeling that no one gets you, nobody understands you.

From this conceptualization, practitioners distinguish between social connection and meaningful social connection.

#### Loneliness Prevalence

From these conceptualizations, practitioners recognize loneliness as fundamentally about meaningful social connection rather than social isolation. Practitioners consistently report significant prevalence rates of loneliness concerns in their digital mental health practice. As per practitioner 4:

Probably 80%‐90% of my clients will feel some degree of loneliness*.* I think certainly it’s more prevalent with the younger clients.

This stark anecdotal statistic highlights the perceived prevalence of loneliness and the need for practitioners to be able to identify and develop strategies to reduce loneliness. Furthermore, the identification of young people presenting with loneliness concerns more frequently than older clients highlights the relevance of loneliness for practitioners working with younger demographics. Practitioner 4 uses the phrase “some degree of loneliness,” which reveals the nuanced nature of loneliness in which there is a spectrum of loneliness severity.

Practitioner 7 gives an overview of loneliness prevalence across the digital mental health platform Kooth:

[Loneliness] almost inevitably seems to come up nowadays, and I say even as if that’s a surprise [...] The young people, particularly, who appear to be quite well connected socially and that ([Loneliness]) can be a great cause of anxiety for them.

The consistency of high prevalence rates across different digital mental health contexts suggests that loneliness represents a fundamental concern requiring practitioner attention.

Practitioner 2 further corroborates this prevalence concern:

You will find that [Loneliness] will come up with most, if not all of our service users at some point

as does practitioner 7:

Just about every client I work with at some level that comes in, [experiences loneliness], whether it’s all the time or just some of the time.

Practitioners have identified the significant prevalence of loneliness concerns across therapeutic roles, suggesting that loneliness is not limited to specific client populations or therapeutic contexts.

### Theme 2: The Contextual Factors and Causes of Loneliness

#### Overview

Theme 2 discusses the contextual causes across environmental and social experiences that perpetuate loneliness. It is notable that the identified factors are intrinsically linked to one another in a homogeneous experience rather than as defined units of environmental context. Two branches of environmental factors appear: the “social environment” of the individual and the “physical environment” in which they live. However, it is often difficult to unravel this combination of lived experience.

#### Transition, Loss, and Loneliness

Life transitions encompass dynamic changes that can break down established support systems and social connections and are an example of the combination of the social and environmental contexts that lead to loneliness. Life transition is a broad term that can encompass experiences from developmental milestones such as leaving home to attend university to less predictable life events, including the breakdown of a relationship or job loss.

The impact of people moving to university was a frequent contextual instigator of loneliness (likely because of the age of clients a majority of the practitioners work with). Practitioner 9 describes:

When the students first go to university, they feel very, very lonely. It’s been a big change for them.

Practitioner 8 elaborates on this experience: “University students and those that have moved away [...] it comes across as a loss,” in terms of both the environment with which they are familiar and the social connections that were severed. This increase in loneliness as students move to university is prevalent across intervention types, and the practitioners identified several reasons for this experience, particularly change and transition from one social group to another. Practitioner 7 contextualizes this impact on social resources:

I would say almost inevitably, even if someone really wants to be going to uni and they’re excited about it, the reality is, so much changes. Similarly, when you leave [University], everything you’re sort of like resources change overnight.

This idea of change and transition is also highlighted by practitioner 6:

I’ve seen a massive influx in the last couple of weeks in terms of universities starting and the number of service users, mainly adults, presenting with loneliness around adapting to that change of routine.

Life transition is not limited to a physical move of location, but can also be linked to the change or breakdown of a romantic or familial relationship. Practitioner 8 describes,

They feel lonely because they’ve left a relationship or a relationship’s broken down [...] They lose themselves because their partner is essentially their whole life.

Practitioner 1 expands on this from their experience as a grief counselor, identifying how this does not relate only to romantic relationships that have broken down or finished, but also in a time of death:

The [Loneliness] which is caused by the grief and the sense that the person is rendered dysfunctional by the grief.

Describing the isolation during grief, they say, “the grief and the type of loss the person is experiencing puts them in a box of isolation,” while also linking grief to a social disconnection from friends when experiencing grief from loss:

there is no one or there are only, a very tiny, tiny number like one or two, max three people with whom they can say they can tell it like it is.

These observations illustrate how external loss can alter entire relational contexts, making previously available forms of emotional expression and social connection inaccessible.

This contextual understanding has implications for intervention approaches; rather than focusing primarily on individual social development, these findings suggest that loneliness during transitions may be better addressed through environmental scaffolding, which supports individuals in developing new relational patterns that fit their changed circumstances.

#### Stigma and Social Barriers

Stigma and social barriers emerged as fundamental drivers of loneliness, with practitioners consistently identifying how social exclusion compounds the emotional impact of the concern. Rather than viewing loneliness as a purely emotional state, practitioners identified how loneliness arises through broader patterns of social marginalization and cultural rejection.

Practitioner 4 identified this distinction between individual experience and societal exclusion, explaining how minority status within a cultural environment creates vulnerability to loneliness:

Feeling disconnected from the group because, actually their way of being just doesn’t fit with the environment that they’re in [...] Often times people kind of get left out.

This conceptualization moves beyond individual deficits to examine how social structures themselves generate isolation for those who deviate from dominant social norms. Practitioner 4 elaborated on this structural dimension:

I think there’s loneliness, which is almost the person’s internal felt sense of the experience. But also there’s exclusion which I think comes from society in general.

This conceptual disconnection varies depending on the environment or social norm to which individuals feel unable to conform.

Cultural and religious differences constituted a source of loneliness. Practitioner 3 described how clients from minority backgrounds experienced isolation due to lack of cultural recognition:

Feeling isolated because of their faith or their cultural beliefs: 'I’m really lonely because I don’t have anyone who’s like me.

This form of loneliness presents a sense of cultural misunderstanding.

The role of perceived identity and loneliness was evident in practitioners’ discussions of sexuality, gender, and the impact of heteronormative expectations. Practitioner 4 described how societal norms about gender and sexuality create exclusionary experiences:

And I guess you see that in things like heteronormativity and kind of the cisgender expectations that are put on people to [...] look a certain way or present a certain way, whereas actually, who’s to say that’s the right way?

This observation highlights how loneliness emerges not from an individual’s ability to form social bonds, but from social expectations and norms that marginalize those who cannot or will not conform.

Practitioner 9 gave further insight into the impact of not being accepted by family or being able to find a community in which individuals feel included because of their sexual orientation:

“If either their family aren’t accepting [of them being gay] or they’re somewhere that where they haven’t got good people around them, where they’re not accepting [...] can lead to so many issues and them [...] just spending time on their own and not going anywhere and becoming really lonely.”

These observations provide further context for both the emotional experience of loneliness of not being accepted and the specific contextual consequence of being othered due to sexuality or gender. The persistence of such exclusion, despite broader social claims of acceptance and progress, was emphasized by practitioner 8, who noted the disconnect between societal expectations and lived reality:

I think there’s a sense of shame very often even, and I find it quite sad that even today in this time of the world, when we think that it may be more acceptable to have difference of any kind. But the reality, the lived reality for a lot of people is not that because they’re living in their small personal culture, families who may or may not accept them.

These findings emphasize that loneliness cannot be understood solely as an individual psychological experience but must be recognized as being shaped by broader social inclusion and exclusion based on identity, culture, and conformity to dominant norms.

#### The Reduction in Social Resources and Increased Cost of Living

The reduction in social resources provided for both young people and adults is a significant factor in the increase in loneliness, as identified by the practitioners. According to practitioner 2:

I think the general theme for me would be the lack of opportunity [...] to connect with people because when I was a kid, there was youth clubs [...] I went to the library regularly; three of those local libraries now have closed down.

The reduction in resources such as social spaces may make it difficult for young people to connect with others.

The quality of social resources does not only affect young people. Practitioner 5 identifies the increased cost of living and reduced funding for community services and support:

There is also the cost of living [...] the pandemic that forced people, who perhaps maybe have problems in the first place, to go inward as well. In our [area] as well, community services are closing down.

The impacts of a struggling economy can make it challenging for people to go out or engage socially.

This combined effect compounds as resources become less accessible, creating fewer opportunities for organic social connection. When contextualized around deprivation, practitioners identified the stigmatizing experience associated with accessing social support, which may limit engagement with available resources. Practitioner 4 highlights this:

I think any kind of deprivation often is associated with some degree of loneliness, and I think it thinking about my NHS work actually where people generally I see because they can’t afford to have private therapy. And a lot of those clients will be accessing things like food banks or involved in social services.

This summary of broad client concerns identifies how issues are often faced in tandem; those who cannot afford to buy their food are probably not able to pay for private therapy. Practitioner 4 further identified the stigmatizing nature of this experience:

Social housing definitely faces a huge amount of challenges that they face day-to-day and I think a lot of that kind of there’s the stigma, but also I think there’s a lot of embarrassment quite often [...] exacerbates the loneliness. So it’s kind of the shame of being on benefits or the shame of accessing a food bank which often is through no fault of their own, it’s just circumstances have led them to that point in life.

This reveals another stigmatized experience that exacerbates feelings of shame and loneliness.

The stigma and contextual factors contributing to loneliness have profound implications for therapeutic communication. Given the shame associated with acknowledging loneliness directly, practitioners described learning to recognize alternative language patterns through which clients express these concerns.

### Theme 3: The Expressions and Language of Loneliness

#### Overview

Theme 3 captures how practitioners recognize loneliness through subtle linguistic cues and expressions rather than direct disclosure [47]. This theme encompasses practitioners’ understanding of the various ways clients communicate loneliness without explicitly saying “I am lonely.”

Practitioners described indirect disclosure, identifying typical characteristics of language clients use to describe their emotions. Practitioners identified differences in the way older adults express loneliness compared with young people, and that some clients do not always have the language to express loneliness. Practitioner 3 describes this:

They have the feeling but they haven’t got the language to go with the feeling, to understand what it is that’s going on. And then once they’ve kind of got some language, it’s almost like, well, how do we understand that feeling and what it means to them?

The limitation of appropriate language affects disclosure, as clients may feel isolated without being able to name or explain their experience, creating a barrier to self-understanding and support.

When analyzing why indirect disclosure is so prevalent, the shame and stigma associated with loneliness emerged. Practitioners explained that clients often give the air of “its only loneliness” (practitioner 7), and that it is not a significant issue that needs addressing. This is combined with the shame of being lonely, of having “no friends to talk to,” or as practitioner 5 explains,

a lot of the time I will pick up on real shame, real stigma and this idea of [...] if you're lonely, you're almost like Billy no mates.

While the stigmatized nature of loneliness plays a role in its indirect disclosure, the language used to express loneliness varies based on the contexts driving the loneliness and the age of the client.

#### Indirect Expressions of Loneliness

Practitioners identified the frequency of indirect loneliness expressions. Practitioner 9 explains that

there isn’t often that someone will just come out and say ‘I’m lonely.’

Similarly, practitioner 5 explained:

I’ve not had a client in the last few years that’s actually turned around and said ‘I’m lonely ever.

The lack of direct loneliness disclosure may reflect the broader experience of loneliness and show how loneliness is often the result of another contextual experience, especially not feeling that one has genuine connections with others. Practitioner 4 explains that

often times [a client] will use quite broader terms such as ‘I don’t feel like I have anyone to talk to’ [...] ‘but I don’t feel like anyone understands me’ or ‘I don’t feel like that my actual friends.’

Practitioners described how proxy terms or “higher level” language are used to approach the concern of loneliness. Practitioner 7 explains:

[A client says] ‘I’m feeling really depressed.’ It’s like, well, what does depressed mean for you, actually? What does that actually mean, what happens when you’re feeling that rather than just accepting that as a term?

Practitioner 2 furthers explains “[Depression] has become quite a big umbrella word,” which takes on a more general meaning of low mood. Many of the practitioners identified the use of clinical language to describe feelings of loneliness, which starts with the term “depressed,” and then the practitioner is able to identify it as stemming from a feeling of loneliness rather than the experience of depression.

When higher-level clinical language is used, practitioners often have to unpack these terms to understand what the client is attempting to express. Practitioner 2 explains:

[A client] will often use the word depressed, I think sometimes without fully understanding as to where that word’s coming from [...] actually it’s low mood, it’s sadness, it’s feelings of isolation or lacking in something in some way of comfort and connection.

Practitioner 7 gives examples of specific words that clients have used:

Service users might come with kind of overarching themes of what are feel ‘depressed,’ or ‘I feel anxious,’ but when they dig in a little bit [...] other factors or kind of causes or feelings might appear.

While these feelings are no doubt valid, practitioners must differentiate between clients experiencing depression or anxiety and those using familiar mental health terms to express underlying concerns.

There are several reasons why loneliness may be disclosed using indirect language. Practitioner 7 notes the idea of loneliness often being obfuscated by the client because they feel that loneliness is a “lesser” issue:

It’s partly the shame, but I think some feel [lonely] is almost too simplistic a word like it should be something more complex than this because the feeling of loneliness can be really it can be a really desolate feeling. So there must be something more to it than just loneliness.

This highlights how loneliness occupies a marginal position within contemporary mental health nomenclature while also remaining stigmatized. While depression and anxiety have become embedded in everyday psychological vocabulary, loneliness lacks equivalent prevalence, and in turn, clients may be using more accessible mental health terminology. While potential proxy terms and disclosure strategies have been identified, it is worth noting that loneliness can co-occur with depression and other mental health concerns. This analysis focuses on instances where practitioners identified that depression-related language masked concerns of loneliness.

#### Age-Related Differences in Expression

Evaluating the differences in loneliness expression can provide a more nuanced understanding of loneliness across age groups and facilitate further examination of the causes of loneliness.

Older people may use direct language, which practitioner 7 described as “traditional” or “less technical” language. Practitioner 1 gave examples used during times of grief such as “dark” and “I don't see the point,” suggesting that their loneliness related to absence or meaninglessness.

Younger children use basic descriptive language but with the consistent avoidance of loneliness terminology present in adults. As practitioner 2 explains, “feeling low.” This language can change considerably across age, as practitioner 2 identifies:

Compared with teenaged clients will be a bit more elaborate, ‘withdrawn,’ *‘*I feel misunderstood*,’* ‘I feel misinterpreted*’* or ‘I don‘t feel heard.’

These findings link to social acceptance, as practitioner 6 notes:

I don’t feel like I’m matter” or “I don't feel I'm good enough

or practitioner 9:

I can’t make any friends. No one seems to like me.

Teenage expression becomes more socially nuanced and metaphorical, reflecting social complexity, with practitioner 9 also identifying expressions such as “feeling lost” or “helpless,” relating loneliness to social experiences. Across the age ranges, metaphorical expressions are consistently present. Practitioner 4 identified terms such as “hollow,” “empty,” and “desolate” as often used by clients to describe feelings of loneliness.

Highlighting the contextual nature of loneliness, younger users often identify concerns around school, as discussed by practitioner 3:

Within the younger users it seems to be a lot more centered around friendships and like school

and practitioner 2:

The majority of your external interaction outside of your family come from those that you interact with in school education.

This suggests that there are consistent experiences that serve as potential catalysts for loneliness, as identified in Theme 2 for adults. School-based loneliness occurs when young people feel othered. Practitioner 2 expands upon not feeling a meaningful connection and how this can affect young people:

If you don’t feel like you’re connecting with those around you, it could be that you’ve got micro interests, your interests that are in this very small bubble others don’t actually share.

Age-related differences in loneliness expression reflect how individuals develop a self-descriptive vocabulary as they navigate increasingly complex social environments, yet avoid direct loneliness terminology. While children use basic descriptive language and teenagers use more socially aware terminology, both groups avoid explicit self-disclosure. Similarly, older adults may use more traditional language but still rely on proxy terms. Regardless of age-related expression, this avoidance reinforces loneliness as a stigmatized experience that requires careful therapeutic consideration across all client demographics.

### Theme 4: The Co-Occurrence of Loneliness and Mental Health Concerns

#### Overview

Theme 4 evaluates the co-occurrence of mental health concerns and loneliness. While clinical language may be used as a proxy for loneliness concerns, it is also valuable to evaluate practitioners’ understanding of the specific relationship between mental health concerns and loneliness. Particularly, this theme examines the impact of severe mental health conditions or presenting issues, the relationship identified by practitioners, and broader social consequences for those experiencing mental health challenges.

#### Pathways to Severe Mental Health Issues

Feelings of invalidation were identified as a driver of severe mental health concerns, which both result from and contribute to loneliness. Practitioner 4 gives an example of the invalidation that arises from exhibiting traits of more severe mental health disorders:

There’s a lot of invalidation about their experience. They’re ‘too upset’ [...] including the systems that in theory are there to support them, quite often, do invalidate, to kind of add to that disconnect.

This shows a link between stigmatized experiences that lead to feeling invalidated and experiences of loneliness. Practitioner 9 elaborates on this link:

All mental health disorders really can lead to [Loneliness] and the people that are, [...] very traumatised as well, people that have had really invalidating, [...] upbringings where the parents haven’t been good.

This further elucidates the connection between poor social support, stigma, and loneliness.

Loneliness can intensify presenting mental health concerns. Practitioner 9 described the relationship between loneliness and mental health severity as follows:

The impact of loneliness on people who are already in the severe category [of mental health concern] [...] the potential impact of their becoming lonely and the risk to life are so significant.

This is a stark insight into the consequences of loneliness combined with severe mental health issues. Practitioner 3 describes a potential progression from social issues such as friendship problems or low self-esteem to “higher presenting risks” with “Friendship issues or some self-esteem issues lead[ing] to higher presenting risk.” This progression suggests escalation toward self-harm or suicide risk. This establishes a need for intervention when the presenting risk is high, as practitioner 3 again identifies,

it can be quite challenging when the young person is quite focused, almost quite fixated on like these feelings almost and their perception of the situation.

This highlights the pathway through which higher-risk issues may result in severe consequences while also causing experiences of loneliness.

#### Loneliness as a Consequence of Specific Mental Health Concerns

While depression and anxiety have become less stigmatized, the impact of less common concerns can lead to an increase in loneliness due to their impact on the individual’s behavior, in part due to these issues being still stigmatized. Practitioner 9 gives specific examples of psychosis and personality disorders:

Major mental health illnesses like psychosis, [..] and personality disorders are still a massive stigma where people, just don’t want people to know but because their symptoms are so intense because they’ve [people with personality disorders] had so much abuse and trauma in the past. And the only way they know how to get a connection is by very extreme behaviours.

This quote identifies how major mental health concerns, which have intense symptoms stemming from trauma, result in the individual resorting to risky behaviors to facilitate a connection.

This isolating behavior can stem from two routes. First, there is the emotional impact of stigma and shame. As practitioner 9 explains:

The shame and everything that comes with [being manic], often they’re more prone to isolating themselves anyway and becoming lonely because they’re depressed.

Self-isolation arises from feelings of embarrassment or fear of judgment, leading to social withdrawal. Second, isolation can result directly from the symptoms of the mental health condition itself. In the case of schizophrenia, practitioner 9 describes:

with schizophrenia that are so paranoid because of their illness [...] some of them will only accept that absolute bare minimum [...] some of them, they won’t even dare put the TV on because they think people are communicating with them through the TV. So you have people like that who are just in their homes just on their own.

In this second pathway, withdrawal is driven by intense paranoia rather than stigma or shame, which limits contact with the outside world.

When the result of a severe mental health issue is hospitalization, the individual’s propensity toward loneliness can increase because of the physical and social separation associated with hospitalization. Practitioners mentioned the impact of specific mental health concerns and loneliness as a co-occurring feedback loop. This consequence was identified by practitioner :

For young people, I find that when they struggle with their mental health, they do tend to lose a lot of friendships sadly and especially if they’ve for example self harmed or attempt to take their own life and they need a hospital admission, a lot of friends then drop them.

Practitioner 6 quotes a service user who was hospitalized:

When I look out of the window of the hospital, all I see is my reflection staring back at me. There’s no one like behind us to show that I'm cared for or I'm looked after.

## Discussion

### Principal Findings

Across all 4 themes, practitioners described loneliness as a complex experience shaped by internal feelings, relational contexts, and broader social structures. The findings show that loneliness is rarely disclosed directly, often masked by proxy language or overshadowed by co-occurring mental health concerns. Furthermore, loneliness is caused by several factors, from life transitions and stigma to the symptoms of severe mental health conditions. This complexity means that loneliness cannot be reduced to the absence of social contact, but must be understood in terms of the quality, meaning, and accessibility of relationships. These observations highlight the importance of practitioner skill in recognizing subtle expressions of loneliness and addressing its psychological and social drivers. The discussion that follows situates these findings within the wider literature, examining how they align with, challenge, and extend existing understandings of loneliness in digital mental health contexts.

This study has further presented the significance of stigma as a facet of loneliness. Stigma operates at two levels within the experience of loneliness: (1) stigma functions as a contextual instigator that increases the propensity for loneliness (through social exclusion and identity-related isolation) and (2) the intrinsic stigma and shame associated with being lonely. This perspective on stigma expands upon previous research identifying the interaction between stigma and loneliness, particularly as practitioners were able to identify concerns through this relationship, suggesting that this can serve as a proxy indicator for identifying loneliness triggers in therapeutic practice.

Practitioner insights about disconnection and a lack of quality social connections aligned with established literature [23,24] and the items in prevailing loneliness measures (particularly the UCLA and DJG Loneliness Scales). This finding resonates with specific concepts from these scales and their conceptualizations of loneliness, in which limited meaningful connections are present for the individual experiencing loneliness (as summarized in Table 4). For example, items 6 and 9 of the UCLA Loneliness Scale (“I have a lot in common with the people around me” and “My interests and ideas are not shared by those around me”) mirror the observations from practitioners that people may feel a lack of connection with others due to surface-level interests. This extends to indirect expression of loneliness; for example, “I don’t feel like they are my actual friends” mirrors item 12 in the UCLA Loneliness Scale: “My social relationships are superficial.”

**Table 4.** Loneliness self-disclosure terms identified in loneliness measurement.

Keyword	Measure item no	Item text
Companion or companionship	UCLA^a^ (item 1)	I lack companionship.
Left out	UCLA (item 10)	I feel left out.
Nobody	UCLA (item 13)	No one really knows me well.
Isolated	UCLA (item 14)	I feel isolated from others.
Withdrawn	UCLA (item 17)	I am unhappy being so withdrawn.
No one to turn to	UCLA (item 20)	There are people I can turn to.
Miss friends	DJG^b^ (items 2 and 9)	(2) I miss having a really close friend. (9) I miss having people around me.
Emptiness	DJG (item 3)	I experience a general sense of emptiness.
Friends	DJG (items 4 and 11)	(4) There are plenty of people I can rely on when I have problems. (11) I can call on my friends whenever I need them.
Trust	DJG (item 7)	There are many people I can trust completely.
Rejected	DJG (item 10)	I often feel rejected.

a UCLA: University of California, Los Angeles Loneliness Scale.

b DJG: De Jong Gierveld Loneliness Scale.

The DJG scale items further reflect practitioner observations about client language patterns. The item “I experience a general sense of emptiness” aligns with practitioners’ reports that clients frequently use metaphorical expressions such as “hollow,” “empty,” and “desolate” to describe their loneliness without using direct terminology. Similarly, the DJG item “I miss having people around me” corresponds to the indirect expressions practitioners identified, such as “I don't feel like I have anyone to talk to.” The DJG item “I often feel rejected” particularly aligns with practitioners’ observations about clients experiencing stigma-based social exclusion, whether due to cultural identity, sexuality, or other marginalizing factors. It should be noted that the items included in these 2 scales were developed in the 1960s and 1970s, suggesting that how individuals describe feelings of loneliness has not changed over time.

Practitioners’ conceptualizations of loneliness align with established theoretical frameworks and measurement approaches. Specifically, practitioner perspectives connect directly with the definition of loneliness as the “subjective perception of a discrepancy between the desired and true social relationships in terms of companionship, connectivity, or intimacy.” [20] Together, these parallels across measurement tools underscore that practitioner perspectives reflect the same relational and emotional dimensions captured in validated loneliness scales, while revealing how these experiences are expressed in therapeutic contexts. This warrants further investigation, as it is unclear whether the coalesced conceptualization is due to the practitioners having been trained on the established scales and concepts, which have shaped their perception, or if their understanding is based on their experience of interacting with and supporting lonely people.

This work also builds upon research on the indirect language of loneliness, demonstrating that expressions of loneliness by clients in traditional therapeutic environments align with those in online and digital interventions [41,48].

These findings extend the findings provided by Verity et al [25], wherein young people described a discrete set of emotions to characterize the experience of loneliness, including “sadness,” “anger,” “emptiness,” “feeling unloved,” and “hopeless.” Furthermore, practitioners noted that clients would disclose feeling disconnected or hopeless about their social situation without directly acknowledging the feeling of loneliness. These feelings were identified as indirect expressions, particularly practitioners reporting clients describing themselves as “depressed” rather than lonely, which aligns with the work by Cacioppo et al [49], which identified some overlap in the experience of depression and loneliness. However, this research identifies that this disclosure of depression could be due to the client’s clinical vocabulary rather than their true experience. More specifically, depression is used as a description of how people feel, which may be true for clients, but this is distinctly different from loneliness as a specific feeling related to the lack of social connection as established by Cacioppo et al [49] and Weiss [50].

This analysis provides novel perspectives on the specific ways in which loneliness is elicited by different age groups, identifying a previously undocumented developmental progression in loneliness language. The language used by older people is often more direct and “traditional” compared to the clinical terminology that has become embedded in young people’s emotional vocabulary, suggesting that mental health literacy may incongruously create new barriers to authentic emotional expression.

The insights reveal how loneliness impacts individuals’ approaches to therapeutic disclosure in digital intervention environments. These findings extend existing loneliness measurement theory by highlighting the value of loneliness measurement tools that do not use direct loneliness questions, such as “How often do you feel lonely?” Rather, these findings validate the effectiveness of indirect assessment approaches while providing insight into their efficacy, due to interpersonal stigma preventing direct therapeutic disclosure.

The contextual triggers of loneliness identified by practitioners were numerous, particularly affecting those who have minority status within a dominant culture and face challenges in conforming to social norms because of their cultural background. This type of stigma can lead to feeling disconnected from the broader culture that surrounds them. Other specific examples include lesbian, gay, bisexual, transgender, and queer or questioning plus or neurodiverse populations [10,31], whose experiences are less understood or who are actively othered by their community.

The significant impact of life transitions identified by practitioners aligns with Lim et al’s [8] assessment of loneliness triggers, where life transitions precede and instigate loneliness. Practitioners identified triggers such as moving away from home [24], divorce (the breakdown of a relationship) [51], or the death of a spouse (with the associated grief and loss) [24].

The practitioners identified reductions in social spaces and government resources combined with an increased cost of living as contributing to loneliness through limitations in social activities due to cost and through the stigma of having to engage in social support; this aligns with and expands on the findings of Cheetham et al [52]. These factors build upon previous research identifying the contextual nature of loneliness while also providing evidence that these elements are consistent in online environments.

While the contextual drivers remain consistent among practitioners in traditional face-to-face interventions and those who practice online, novel insight has been gleaned into the way in which clients, specifically younger clients, express loneliness. Furthermore, regardless of the environment, loneliness is still expressed indirectly through metaphors and similes to minimize the perceived stigma of loneliness.

### Limitations

Recruitment challenges within the specialized population of digital mental health practitioners limited the sample size to 9 participants. The sample was mostly female and entirely White British (where reported), potentially limiting the generalizability of findings across diverse practitioner and client populations. However, the achieved sample demonstrated strong conceptual diversity across practitioner roles and platform types, supporting the credibility of identified themes.

Following recommendations for pragmatic saturation assessment [53], saturation was evaluated based on the sufficiency of data to address research objectives. The central patterns of loneliness expression, contextual causes, and practitioner recognition had reached sufficient depth for meaningful analysis, though additional participants might have provided further nuance.

While this study identified how loneliness is expressed indirectly in therapeutic contexts, questions remain about how digital interventions can effectively address loneliness given these indirect disclosure patterns. The practitioners’ perspectives suggest that understanding service users’ underlying needs may be crucial for developing effective interventions. This provides the foundation for examining how frequently loneliness-related concerns appear among the broader needs of digital mental health service users.

### Conclusion

This study provides a theoretical contribution to the literature, collating and synthesizing practitioners' insights. The practitioners identified multiple stigmatizing experiences as contextual drivers of loneliness, highlighting how loneliness emerges not only from individual factors but from broader patterns of social exclusion and marginalization. For therapeutic practice, these insights suggest that practitioners can use awareness of stigmatizing experiences as potential indicators when assessing loneliness risk. The presence of these contextual patterns was consistent across digital therapeutic environments, providing a foundation for developing more targeted interventions that address both the emotional experience of loneliness and its underlying social drivers.

## Abbreviations

ACTO: Association for Counselling and Therapy Online
DJG: De Jong Gierveld Loneliness Scale
GP: general practitioner
TA: thematic analysis
UCLA: University of California, Los Angeles

## References

[1] Public opinions and social trends, great britain: July 2024. Office for National Statistics (ONS).

[2] (2023). The state of loneliness 2023: ONS data on loneliness in Britain June 2023. https://www.campaigntoendloneliness.org/wp-content/uploads/The-State-of-Loneliness-2023-ONS-data-on-loneliness-in-Britain.pdf.

[3] Perissinotto CM, Stijacic Cenzer I, Covinsky KE (2012). Loneliness in older persons: a predictor of functional decline and death. Arch Intern Med.

[4] Ruan J, Xu YM, Zhong BL (2023). Loneliness in older Chinese adults amid the COVID-19 pandemic: prevalence and associated factors. Asia Pac Psychiatry.

[5] Caple V, Maude P, Walter R, Ross A (2023). An exploration of loneliness experienced by people living with mental illness and the impact on their recovery journey: an integrative review. J Psychiatr Ment Health Nurs.

[6] de Jong-Gierveld J (1987). Developing and testing a model of loneliness. J Pers Soc Psychol.

[7] Russell DW (1996). UCLA Loneliness Scale (Version 3): reliability, validity, and factor structure. J Pers Assess.

[8] Lim MH, Eres R, Vasan S (2020). Understanding loneliness in the twenty-first century: an update on correlates, risk factors, and potential solutions. Soc Psychiatry Psychiatr Epidemiol.

[9] Ludwig KA, Brandrett B, Lim MH, Mihas P, Penn DL (2022). Lived experience of loneliness in psychosis: a qualitative approach. J Ment Health.

[10] Barreto M, van Breen J, Victor C (2022). Exploring the nature and variation of the stigma associated with loneliness. J Soc Pers Relat.

[11] Reupert A, Gladstone B, Helena Hine R (2021). Stigma in relation to families living with parental mental illness: an integrative review. Int J Ment Health Nurs.

[12] Balcombe L, De Leo D (2022). Evaluation of the use of digital mental health platforms and interventions: scoping review. Int J Environ Res Public Health.

[13] Braciszewski JM (2021). Digital technology for suicide prevention. Adv Psychiatry Behav Health.

[14] Iorfino F, Cross SP, Davenport T (2019). A digital platform designed for youth mental health services to deliver personalized and measurement-based care. Front Psychiatry.

[15] Schueller SM, Torous J (2020). Scaling evidence-based treatments through digital mental health. Am Psychol.

[16] Milligan G, Bernard A, Dowthwaite L (2024). Developing a single‐session outcome measure using natural language processing on digital mental health transcripts. Couns and Psychother Res.

[17] Davis HA, Kells M, Todorov S, Kosmas J, Wildes JE (2023). Comorbid eating, depressive, and anxiety psychopathology is associated with elevated shame in women with food insecurity. Int J Eat Disord.

[18] Park C, Majeed A, Gill H (2020). The effect of loneliness on distinct health outcomes: a comprehensive review and meta-analysis. Psychiatry Res.

[19] Qualter P, Vanhalst J, Harris R (2015). Loneliness across the life span. Perspect Psychol Sci.

[20] Hawkley LC, Cacioppo JT (2010). Loneliness matters: a theoretical and empirical review of consequences and mechanisms. Ann Behav Med.

[21] Hawkley LC (2022). Loneliness and health. Nat Rev Dis Primers.

[22] Victor CR, Yang K (2012). The prevalence of loneliness among adults: a case study of the United Kingdom. J Psychol.

[23] Martin K, Wood L, Houghton S (2014). “I don’t have the best life”: a qualitative exploration of adolescent loneliness. J Child Adolesc Behav.

[24] Fardghassemi S, Joffe H (2022). The causes of loneliness: the perspective of young adults in London’s most deprived areas. PLoS One.

[25] Verity L, Schellekens T, Adam T (2021). Tell me about loneliness: interviews with young people about what loneliness is and how to cope with it. Int J Environ Res Public Health.

[26] BARRY MJ Jr (1962). Depression, shame, loneliness, and the psychiatrist’s position. Am J Psychother.

[27] Cooper F (2024). “Solitude is not thrust upon any lovable person”: loneliness, shame, and the problem (of) personality. J Psychosoc Stud.

[28] Paton E, Jones EP, Peprah J, Benson M (2024). Our words matter: finding consensus on evolving and personal language around suicide, mental health concerns and alcohol and other drug use. Media Int Aust.

[29] Klin A, Lemish D (2008). Mental disorders stigma in the media: review of studies on production, content, and influences. J Health Commun.

[30] Zhang H, Firdaus A (2024). What does media say about mental health: a literature review of media coverage on mental health. Journal Media.

[31] Dahlberg K (2007). The enigmatic phenomenon of loneliness. Int J Qual Stud Health Well-being.

[32] Asher SR, Wheeler VA (1985). Children’s loneliness: a comparison of rejected and neglected peer status. J Consult Clin Psychol.

[33] Madsen KR, Tjørnhøj-Thomsen T, Jervelund SS, Qualter P, Holstein BE (2021). Lonely, but not alone: qualitative study among immigrant and native-born adolescents. Int J Environ Res Public Health.

[34] Abplanalp SJ, Green MF, Wynn JK (2024). Using machine learning to understand social isolation and loneliness in schizophrenia, bipolar disorder, and the community. Schizophr.

[35] Mann F, Wang J, Pearce E (2022). Loneliness and the onset of new mental health problems in the general population. Soc Psychiatry Psychiatr Epidemiol.

[36] Mushtaq R, Shoib S, Shah T, Mushtaq S (2014). Relationship between loneliness, psychiatric disorders and physical health ? A review on the psychological aspects of loneliness. J Clin Diagn Res.

[37] Gasull-Molinera V, Khan KS, Núñez Núñez M, Kouiti M (2024). The impact of loneliness on mental and physical health outcomes: an umbrella review. Semergen.

[38] Birken M, Chipp B, Shah P (2023). Exploring the experiences of loneliness in adults with mental health problems: a participatory qualitative interview study. PLOS One.

[39] Stefanidou T, Wang J, Morant N, Lloyd-Evans B, Johnson S (2021). Loneliness in early psychosis: a qualitative study exploring the views of mental health practitioners in early intervention services. BMC Psychiatry.

[40] Dobarrio-Sanz I, Ruiz-González C, Fernández-Sola C, Roman P, Granero-Molina J, Hernández-Padilla JM (2021). Healthcare professionals’ perceptions of loneliness amongst older adults: a qualitative study. Int J Environ Res Public Health.

[41] Verity L, Yang K, Nowland R, Shankar A, Turnbull M, Qualter P (2024). Loneliness from the adolescent perspective: a qualitative analysis of conversations about loneliness between adolescents and childline counselors. J Adolesc Res.

[42] Jovicic A, McPherson S (2020). To support and not to cure: general practitioner management of loneliness. Health Soc Care Community.

[43] Lawn S, Kaine C, Stafrace S (2023). Why talking about loneliness matters to the mental health of consumers and to the work of the psychiatrist. Aust N Z J Psychiatry.

[44] Alhojailan MI (2012). Thematic analysis: a critical review of its process and evaluation. West East Journal of Social Sciences.

[45] Saunders B, Sim J, Kingstone T (2018). Saturation in qualitative research: exploring its conceptualization and operationalization. Qual Quant.

[46] Braun V, Clarke V (2006). Using thematic analysis in psychology. Qual Res Psychol.

[47] Solano CH, Batten PG, Parish EA (1982). Loneliness and patterns of self-disclosure. J Pers Soc Psychol.

[48] Heu LC, Hansen N, van Zomeren M (2021). Loneliness across cultures with different levels of social embeddedness: a qualitative study. Pers Relatsh.

[49] Cacioppo JT, Hawkley LC, Thisted RA (2010). Perceived social isolation makes me sad: five year cross-lagged analyses of loneliness and depressive symptomatology in the Chicago Health, Aging, and Social Relations Study. Psychol Aging.

[50] Weiss R (1975). Loneliness: The Experience of Emotional and Social Isolation.

[51] Schobin J (2022). Loneliness and emancipation: a multilevel analysis of the connection between gender inequality, loneliness, and social isolation in the ISSP 2017. Int J Environ Res Public Health.

[52] Cheetham M, Moffatt S, Addison M, Wiseman A (2019). Impact of Universal Credit in North East England: a qualitative study of claimants and support staff. BMJ Open.

[53] Hennink M, Kaiser BN (2022). Sample sizes for saturation in qualitative research: a systematic review of empirical tests. Soc Sci Med.

